# Transcatheter Folded Valve Placement Within a Patent Ductus Arteriosus for Pulmonary Hypertension

**DOI:** 10.1016/j.jaccas.2026.108137

**Published:** 2026-05-04

**Authors:** Eli S. Fredman, Hadeel Allam, Nidhy P. Varghese, Daisuke Kobayashi, Manish Aggarwal, R. Mark Grady, David Balzer

**Affiliations:** a Division of Pediatric Cardiology, Washington University School of Medicine, St Louis Children's Hospital, St Louis, Missouri, USA; b Division of Pediatric Pulmonology, Department of Pediatrics, Baylor College of Medicine, Texas Children's Hospital, Houston, Texas, USA

**Keywords:** Melody valve, patent ductus arteriosus, pediatric cardiology, pulmonary arterial hypertension, reverse potts shunt, transcatheter valve implantation

## Abstract

**Background:**

Severe pulmonary arterial hypertension (PAH) with bidirectional patent ductus arteriosus (PDA) presents a challenge, as ductal closure is contraindicated.

**Case Summary:**

A 14-year-old boy with severe PAH and bidirectional PDA experienced exercise intolerance. He underwent transcatheter placement of a 20-mm folded Melody valve within the native PDA. The procedure eliminated diastolic left-to-right shunting while preserving systolic right-to-left shunting. At 1-year follow-up, he demonstrated durable unidirectional shunting and improved to NYHA functional class I status.

**Discussion:**

While surgical valved reverse Potts shunts exist in the literature, adapting a transcatheter valve to create a unidirectional shunt within the native PDA percutaneously is novel. It offers a less invasive alternative for managing suprasystemic PAH.

**Take-Home Messages:**

Transcatheter folded valve placement within a native PDA safely establishes a unidirectional right-to-left shunt. This intervention reduces pulmonary overcirculation while maintaining vital ventricular decompression.

## Visual Summary

**Figure:**
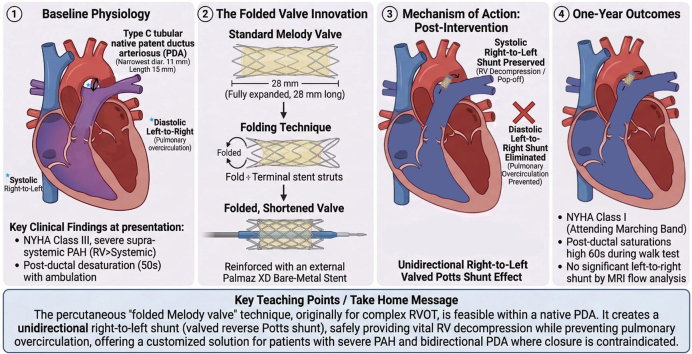

**Visual Summary:**
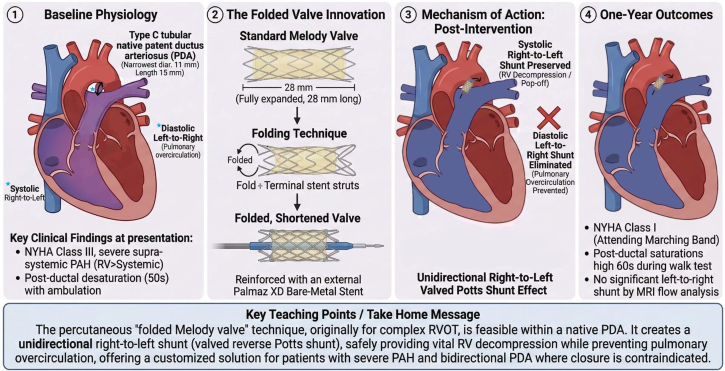
Infographic Detailing the Percutaneous Creation of a Valved Reverse Potts Shunt Using a Folded Melody Valve Within a Native Patent Ductus Arteriosus The summary outlines the patient's baseline physiology, the valve modification technique, the unidirectional hemodynamic mechanism of action, and successful 1-year clinical outcomes. MRI = magnetic resonance imaging; PAH = pulmonary arterial hypertension; PDA = patent ductus arteriosus; RV = right ventricle; RVOT = right ventricular outflow tract.

## History of Presentation

A 14-year-old boy with severe pulmonary arterial hypertension (PAH) associated with a large bidirectional patent ductus arteriosus (PDA) was referred for transcatheter intervention. He had deteriorated to NYHA functional class III status, presenting with worsening fatigue, chest pain, and widening pre- and postductal oxygen saturation splits (postductal saturations dropped into the 50s with ambulation). Physical examination showed increased precordial activity, with a loud S_2_ and no murmur. At rest, his preductal saturations were 97% and postductal saturations were 82%.

**Take-Home Messages**

• Innovative transcatheter techniques, such as the “folded Melody valve,” can establish a unidirectional right-to-left shunt within a native patent ductus arteriosus.
• This customized intervention safely eliminates pulmonary overcirculation while preserving essential right ventricular decompression in patients with severe pulmonary arterial hypertension.

## Past Medical History

Adopted internationally at age 2, he was diagnosed with severe PAH and a large bidirectional PDA at age 6 after presenting with exercise intolerance and failure to thrive. Initial cardiac catheterization revealed a baseline indexed pulmonary vascular resistance (PVRi) of 33 WU·m^2^, which decreased to 24 WU·m^2^ with inhaled nitric oxide (iNO). He was started on upfront combination therapy, including a treprostinil infusion, in hopes of allowing safe PDA closure. Subsequent catheterization at age 11 showed systemic right ventricular (RV) pressure, mean pulmonary artery (PA) pressure of 51 mm Hg, and PVRi of 9.9 WU·m^2^ without response to vasoreactivity testing. Owing to preserved RV function, he was deemed too well for lung transplantation. Prioritizing quality of life over cure, he had been transitioned to oral combination therapy including selexipag before his recent clinical deterioration.

## Differential Diagnosis

Given his progressive fatigue, chest pain, and exertional desaturation, the differential diagnosis included natural disease progression of severe pulmonary vascular disease, worsening right heart failure, or progressive pulmonary overcirculation and volume overload secondary to unrestrictive diastolic left-to-right shunting across the PDA.

## Investigations

Computed tomography angiography showed a tubular PDA (15 mm length; 11 mm narrowest diameter). Three-dimensional modeling of the computed tomography angiography dataset was used to simulate the implant, confirming that the folded valve dimensions would accommodate the native anatomy without impingement on the aorta or branch PAs. Cardiac magnetic resonance imaging demonstrated RV hypertrophy, borderline dilation (RV end-diastolic volume: 107 mL/m^2^), and mildly reduced function (RV ejection fraction: 44%). Flow analysis revealed predominantly systolic right-to-left and diastolic left-to-right PDA flow. The combined branch PA net flow was 54 mL, with 27 mL supplied in diastole by the PDA, indicating that half of the PA flow originated from the left-to-right PDA flow. Left ventricular systolic function was normal (ejection fraction: 57%).

**Figure 1:**
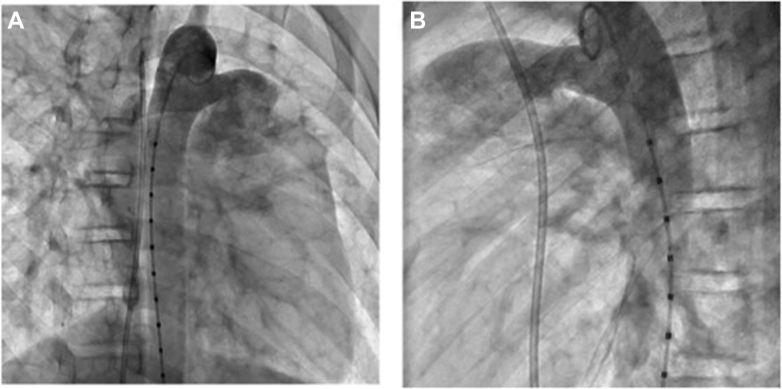
Preintervention Angiographic Image Frontal camera with (A) slight right anterior oblique angulation and (B) lateral views of an aortogram through a 4-F pigtail catheter showing a large, type C, tubular patent ductus arteriosus with flow entering dilated pulmonary arteries.

Catheterization under general anesthesia showed slightly suprasystemic RV systolic pressure (86 mm Hg), mean PA pressure of 70 mm Hg, and transpulmonary gradient of 60 mm Hg. Detailed hemodynamics revealed a pulmonary vascular resistance of 10.8 WU and a resistance ratio (Rp/Rs) of 0.89. The cardiac output was 5.1 L/min, with a cardiac index of 4.2 L/min/m^2^. PVRi was elevated at 13 WU·m^2^, with no response to vasoreactivity testing (100% FiO2, 40 ppm iNO) as defined by the modified Barst criteria.^1^ Angiography confirmed a large, type C tubular, bidirectional PDA and dilated branch PAs (Figure 1). The PDA length measured 17.8 mm, the aortic ampulla dimension 15.6 mm, the PA end 14.5 mm, and the narrowest dimension 11.3 mm.

## Management

**Figure 2:**
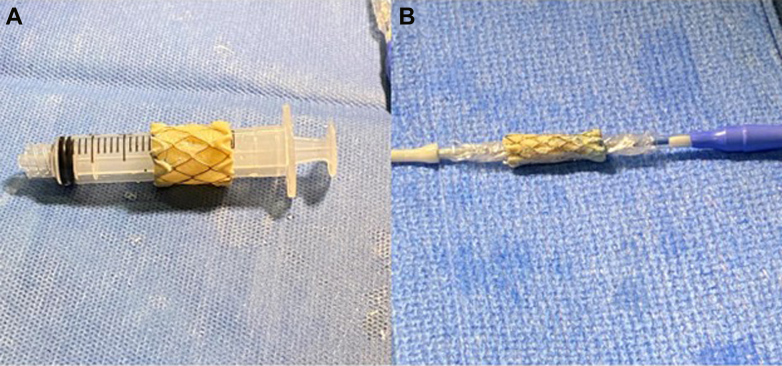
Folded Melody Valve Preparation (A) Distal stent struts of the Melody valve are folded over a 5-mL syringe before crimping the valve. (B) A Palmaz 2510 XD biliary stent placed over the folded Melody valve and the entire ensemble has been crimped on the balloon-in-balloon system.

Standard expansion of a Melody valve (Medtronic) to 18 mm would result in a length of 28 mm, potentially causing obstruction. Therefore, the “folded valve” modification was used. Terminal stent struts were folded inside-out on a 5-mL syringe to shorten the device. The valve stent was then crimped onto a 20-mm Ensemble delivery system (Medtronic). To reinforce radial strength, a Palmaz 2510 XD bare-metal stent (Cordis) was mounted directly over the folded valve (Figure 2).

**Figure 3:**
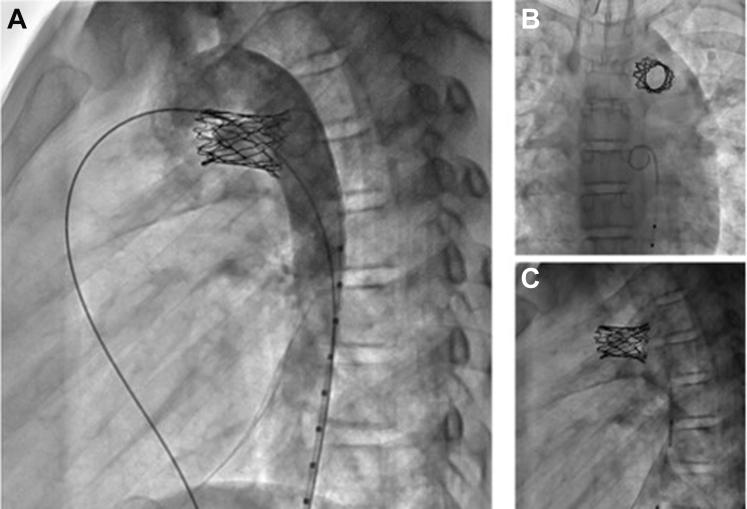
Postintervention Angiographic Images (A) Lateral view showing folded 20-mm Melody valve fully deployed within the patent ductus arteriosus with complete elimination of the left-to-right shunt. (B) Straight anteroposterior and (C) lateral views of the final device positioning at the conclusion of the procedure.

Using a right femoral vein approach, the system was advanced over a 0.035-inch Amplatz Super Stiff wire (Boston Scientific). The valve and stent were delivered simultaneously via a single inflation of inner and outer balloons and postdilated with a 14-2 Atlas Gold balloon (Bard) at 12 atm. After deployment, the mean and diastolic PA pressures dropped, with the mean dropping 15 mm Hg below the systemic mean, driven by the elimination of diastolic left-to-right flow. As intended, the systolic PA pressure appropriately remained at or slightly above systemic levels to permit required right-to-left shunting. Angiography confirmed elimination of diastolic left-to-right flow (Figure 3). There were no complications.

## Outcome and Follow-Up

The patient was discharged on daily aspirin and was advised to maintain lifelong subacute bacterial endocarditis prophylaxis. At the 4-month follow-up, he improved to NYHA functional class II status, with postductal saturations of 88%. Repeat catheterization showed primarily right-to-left shunting at rest. During acute vasoreactivity testing, the decrease in pulmonary vascular resistance diminished the driving force for the right-to-left shunt. Crucially, there was no significant PA saturation step-up despite pulmonary vasodilation, confirming the valve successfully prevented left-to-right shunting. Descending aortic saturations remained lower than ascending aorta saturations, confirming maintained unidirectional right-to-left shunt physiology. Although descending aortography showed mild Melody valve insufficiency, quantitative magnetic resonance imaging flow analysis showed a reduction in diastolic flow fraction from 50% to 22%.

At 1 year postintervention, he is at NYHA functional class I, attending school, and participating in marching band. He remains on triple oral combination therapy. He walks >600 m without symptoms, and preductal/postductal saturations are in the 90s at rest, with postductal saturations in the high 60s on 6-minute walk test.

## Discussion

Severe PAH is a progressive disease with high morbidity and limited treatment options.^2^ Current guidelines recommend pulmonary vasodilators to improve functional class and effort tolerance in patients with Eisenmenger physiology; however, managing a bidirectional PDA remains challenging. Patency causes pulmonary volume overload, while closure is absolutely contraindicated, as it risks acute RV failure. Using vasodilators in the presence of an unrestrictive bidirectional communication presents a therapeutic paradox: Lowering the pulmonary vascular resistance without a fixed anatomic obstruction can inadvertently increase left-to-right shunting, exposing the pulmonary vasculature to increased volume and flow.^3^ This underscores the need for a mechanical strategy that preserves protective right-to-left decompression while eliminating diastolic left-to-right shunting.

To address the limitation of bidirectional flow, the reverse Potts shunt creates a pulmonary artery-to-aorta anastomosis to offload the RV in suprasystemic PAH. Surgical modifications using valved conduits have been introduced to ensure unidirectional right-to-left shunting while preventing systemic-to-pulmonary flow.^4^ Transcatheter valve placement within a surgical nonvalved Potts shunt conduit has also been reported.^5^^,^^6^ Although transcatheter valved shunt creation directly within the native PDA remains outside the scope of current guideline-recommended practices, this mechanical approach provides a tailored, less invasive alternative to high-risk surgical palliation to resolve this hemodynamic paradox.^7^^,^^8^

The present case demonstrates the feasibility of adapting the “folded Melody valve” technique to the native PDA. Originally developed for complex RV outflow tracts, folding the terminal struts shortens the Melody valve sufficiently to fit without obstructing the aorta or PA.^8^ Physiologically, eliminating the diastolic left-to-right shunt removes excess volume returning to the pulmonary circulation, decreasing total pulmonary blood flow (Qp) and overall PA pressures. The systolic PA pressure appropriately remained at or slightly above systemic levels to allow the RV to decompress into the aorta, mimicking the physiology of a reversed valved Potts shunt.

The Melody valve incorporates a platinum-iridium stent with relatively low radial strength. To minimize the risk of stent compression, we reinforced the assembly by mounting an additional bare-metal stent (Palmaz) directly over the folded valve. Intervening on a native PDA requires extreme caution, as balloon dilation carries a critical risk of ductal rupture. The folded Melody valve, despite its lower intrinsic radial strength, provided superior anatomic fit compared with shorter, rigid alternatives such as the Edwards Sapien series, which would have required dangerous overdilation of the 11.3-mm native ductus.

While the 1-year clinical improvements are highly encouraging, continued longitudinal follow-up is essential to evaluate the long-term impact on functional class, right ventricular remodeling, and overall survival.

## Conclusions

Transcatheter placement of a folded Melody valve within a PDA is an effective option in select patients with PAH and bidirectional shunting. This technique reduces pulmonary overcirculation and improves hemodynamics—specifically by eliminating left-to-right diastolic flow and increasing effective pulmonary blood flow—while maintaining a vital pop-off for RV decompression. It offers a viable alternative when surgical or standard transcatheter options are contraindicated.

## Funding Support and Author Disclosures

The authors have reported that they have no relationships relevant to the contents of this paper to disclose.
